# Patient-tailored symptom tracking in endometriosis: a framework to explore disease variability and burden

**DOI:** 10.1038/s41746-026-02965-z

**Published:** 2026-07-17

**Authors:** Tamar Zelovich, Alon Zelovich, Shaked Feiglin, Vered Klaitman-Mayer, Noémie Elhadad, Ronya Rubinstein, Ronit Endevelt, Pinchas Akiva, Maytal Bivas-Benita, Chen Yanover

**Affiliations:** 1 KI Research Institute, Kfar Malal, Israel; 2 Faculty of Health Sciences, Ben Gurion University of the Negev, Beer Sheva, Israel; 3 Maccabi Health Services, South District, Israel; 4 Columbia University Irving Medical Center, Department of Biomedical Informatics, New York, NY USA; 5 School of Public Health, University of Haifa, Haifa, Israel

**Keywords:** Computational biology and bioinformatics, Diseases, Health care, Mathematics and computing, Medical research

## Abstract

Endometriosis is a chronic, estrogen-dependent inflammatory disease characterized by highly individualized, multisystemic symptoms, affecting approximately 10% of females. In this work, we present a long-term prospective study in which 34 participants defined personalized symptom sets and severity scales, tracking them daily for up to 12 months using a custom-developed app. This patient-tailored, high-resolution monitoring revealed substantial heterogeneity in the number of symptoms tracked, with participants tracking a median of 24.5 unique symptoms (interquartile range [19.25, 36.5]). To standardize assessment of system-organ involvement, symptoms were mapped to the MedDRA hierarchy, allowing structured analysis of symptom distribution. Based on these data, we propose a framework for characterizing symptom burden, trajectory, and disease burden, concepts that together capture the variability, systemic nature, and complexity of endometriosis. This personalized approach offers a clearer understanding of the disease experience and lays the groundwork for future tools that may improve communication with healthcare providers and inform more personalized and effective treatment strategies.

## Introduction

Endometriosis is a chronic, systemic, inflammatory and estrogen-dependent gynecological disease that affects about 10% of women of reproductive age worldwide^1–3^. Globally, it is estimated that 170 million women are living with endometriosis^4^. Despite its high prevalence, the average delay from symptom onset to diagnosis, which often requires surgical confirmation, has been estimated at 4–12 years^3,5–8^. Endometriosis is characterized by endometrial-like cells growing outside the uterus, primarily in the pelvic area. These cells respond to hormonal changes, and, similar to menstruation, they may bleed, leading to inflammation, scar tissue formation, and adhesions that bind pelvic tissues and organs^9,10^.

The complexity of managing endometriosis arises from its wide range of symptoms affecting multiple organ systems^11^, with high heterogeneity in symptom presentation among patients^2,12–15^. Symptoms may include severe pain during menstruation and or ovulation, pelvic pain, lower back pain, pain during intercourse, digestive^16–20^ and urinary disturbances, fatigue^12^, radiating leg pain^21–23^, migraines^16,17,24,25^, mood swings, and infertility^26,27^. This variability, along with the fluctuating intensity of symptoms throughout the menstrual cycle, makes disease management particularly challenging, requiring highly individualized treatment approaches^28–32^.

These complexities also create significant barriers to effective patient–physician communication^33^. Many patients struggle to accurately present the full spectrum of their symptoms. Tracking and describing these symptoms over time can be overwhelming, often leading to incomplete reporting or omission of key details. Therefore, it is often difficult to provide a comprehensive picture of a disease that fluctuates over months, leaving patients feeling unable to fully convey the breadth of their condition without fear of being dismissed or misunderstood^34,35^. Physicians, in turn, face additional challenges due to time constraints during consultations, limiting their ability to thoroughly address every symptom reported^36–38^. Without systematic tools to collect and present symptoms and comorbidity data, gaining a comprehensive understanding of the disease remains difficult. This communication gap between patients and physicians^39–43^ can result in inadequate treatment decisions, ineffective disease management^44^, and the potential of worsening symptoms and associated comorbidities^33,45^.

Previous studies have explored the use of digital platforms in endometriosis, highlighting their value in tracking symptoms, improving patient–physician communication, and enhancing disease management^28,34,46–52^. Some platforms allowed tracking of dozens of symptoms and even enabled customized entries, with long-term usage studied across large populations over periods of six months or more. However, even with these technologies, symptom tracking was often based on predefined or semi-customized categories, limiting the ability to fully capture the complexity and individuality of each patient’s experience. Studies with longer durations faced additional challenges, such as significant data gaps due to the lack of mechanisms to encourage consistent symptom tracking^28,34,48–53^.

This study investigates how long-term, personalized symptom tracking can be used to characterize the complexity and multisystemic burden of endometriosis at the individual level. Specifically, we explored symptom heterogeneity across patients, the diversity of organ systems involved, and the extent to which structured symptom classification and longitudinal visualization can capture this complexity. Beyond characterizing symptom heterogeneity, this study aims to provide a conceptual and methodological framework for investigating individualized symptom dynamics and disease burden in endometriosis. We conducted a long-term prospective study involving 34 endometriosis patients who were instructed to log symptoms daily using a custom-developed app. Guided by our research team, each participant defined a personalized set of symptoms and rating scales, enabling individualized longitudinal tracking over time. This approach allowed us to construct a distinct “disease profile” for each individual, reflecting their specific constellation of symptoms, severity, and the systems affected. We further analyzed these data using a system-level classification framework to group symptoms by affected organ systems. This provided a clearer representation of disease complexity and demonstrated how personalized app-based tracking can reveal the systemic burden and heterogeneity of endometriosis. While this study does not assess the impact on patient–physician communication or analyze symptom patterns over time, it establishes a critical foundation for future research in those areas.

## Results

The study cohort included 34 participants. Table 1 summarizes the cohort characteristics. The median participant age was 33 years (interquartile range, IQR, [30.25,36.75]; Fig S1). The median age at first endometriosis symptoms was 14 years [12.75,17], and the median age at diagnosis was 29 years [24,31]. Seventeen participants (50%) had a confirmed diagnosis of adenomyosis, while the remaining 17 either did not have it or were unsure. Two participants (6%) reported known fertility problems, six (18%) reported no impact on fertility, and 26 (76%) reported that they did not know their fertility status at the time of the study. Supplementary Table S1 provides additional cohort characteristics.

**Table 1:** Study cohort characteristics

***N***^a^	34	**Fertility problem**^**b**^	
**Age**^**a**^ [years]		Yes	2 (6%)
When joining the study	33 [30.25, 36.75]	No	6 (18%)
At first period	12 [11.12, 13.75]	Unknown	26 (76%)
At first endometriosis symptom	14 [12.75, 17]	**Adenomyosis**^**b**^	
At endometriosis diagnosis	29 [24, 31]	Yes	17 (50%)
		No	13 (38%)
**BMI**^a^ [kg/m^2^]	21.89 [20.36, 23.41]	Don’t know	4 (12%)
**Family history**^b^		**Diagnostic surgery**^**b**^	
Mom	5 (15%)	No	28 (82%)
Sister	2 (6%)	Yes	6 (18%)
Aunt	5 (15%)	One surgery	5 (15%)
Cousin	2 (6%)	>1 surgery	1 (3%)
Grandmother	2 (6%)	**Menstrual regularity**^**b**^	
Grandma’s cousin	1 (3%)	Regular	31 (91%)
Unknown	22 (65%)	Irregular	3 (9%)

*BMI* body mass index.

^a^Median [IQR].

^**b**^Number of participants (% of the study cohort).

### Study characteristics and symptoms variation

**Fig. 1:**
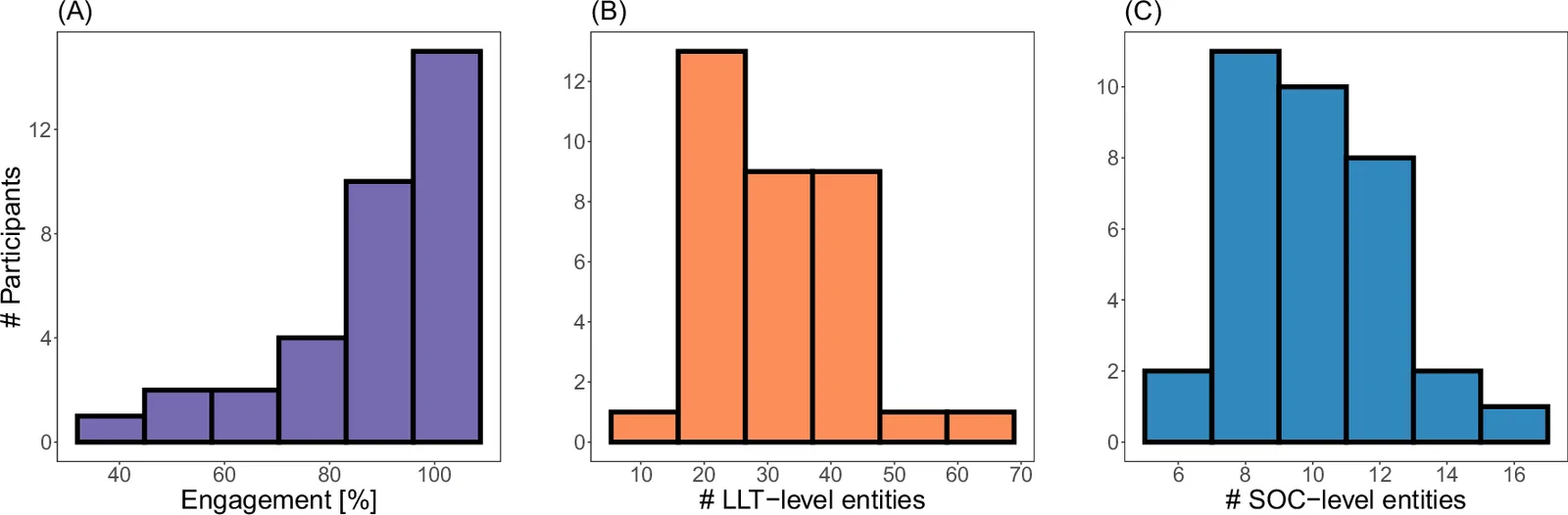
Distribution of a selected set of study-related attributes from Table 2. The y-axis represents the number of participants. Panels illustrate: (**A**) Participant engagement, defined as the ratio of days with reports for at least half of the symptoms to the total days in the study; (**B**) and (**C**) Counts of distinct Lowest Level Terms (LLTs) and System Organ Classes (SOCs), respectively, to which tracked symptoms are mapped.

Table 2 presents statistics of the study attributes, including the average number of reported days and hormonal cycles, as well as characteristics of the tracked symptoms and their hierarchical classification within MedDRA^54,55^. Fig. 1 and Supplementary Figure S2 illustrate the distribution of these attributes, while detailed participant-level data is provided in Supplementary Table S2. As shown in Table 2 and Fig. 1A, the median engagement rate (see Methods for definition) was 95.3% [76.8%,97.4%] and 74% of participants achieved an engagement rate of 80% or higher. Table 2 and Fig. 1B-C also show that the median number of MedDRA’s Lowest Level Terms (LLTs) tracked by participants was 28 [20.2,40.8], affecting 10 [9,12] System Organ Classes (SOCs). In total, participants tracked 228 distinct symptoms, with each participant experiencing a unique set of symptoms, highlighting the complexity and heterogeneity of endometriosis symptomatology. According to the MedDRA hierarchy, these 228 participant-selected symptoms were mapped to 147 LLTs, 123 Preferred Terms (PTs), 112 High-Level Terms (HLTs), 65 High-Level Group Terms (HLGTs), and 17 SOC entries.

**Table 2:** Study Characteristics

Tracked symptoms^a,c^		Tracked cycles	
Number of app symptoms	24.5 [19.25, 36.5]	Number of cycles^c^	5 [3, 7]
Number of LLT entities	28 [20.2, 40.8]	Less than 3^d^	6 (18%)
Number of PT entities	25.5 [20, 36.8]	Three or more^d^	28 (82%)
Number of HLT entities	27.5 [21.2, 38.8]	Cycle length^c^	28.5 [27, 31.1]
Number of HLGT entities	19 [16, 25]		
Number of SOC entities	10 [9, 12]	**Study incompletion reason**^d^	
**Tracked days**^c^		Pregnancy	4 (12%)
Total days in the study	134 [93.5, 218.8]	Personal (layoffs, divorce)	2 (6%)
Engagement [%]^b^	95.3 [76.8, 97.4]	War-related reasons	1 (3%)

^a^Symptoms mapped to MedDRA (excluding General emotional condition, General physical condition, Ovulation test values, Bleeding, medications and well-being related entities).

^b^Percent of days with reports for at least half of the symptoms.

^c^Median [IQR].

^d^Number of participants (% of the study cohort).

Table 3 presents the frequency of SOCs, derived from categorizing the symptoms monitored by participants. For each SOC, the three most frequently tracked LLTs are reported. Because individual LLTs can be linked to more than one SOC, some symptoms, such as dizziness, appear in multiple categories. The number next to each LLT indicates how many participants monitored that particular symptom. The most frequently reported LLTs in our cohort include headache and migraine, fatigue, and low back pain, among others. While LLT-level data highlight specific symptoms, analysis at the SOC level underscores the multisystemic nature of endometriosis, with frequent involvement of the reproductive system and breast disorders, the gastrointestinal system, musculoskeletal and connective tissue disorders, and other systems. A comprehensive list of symptom frequencies, both at the app-defined level and across all MedDRA hierarchy levels, is available in Supplementary Data 1.

**Table 3:** Frequencies of SOCs derived from symptoms tracked by participants

SOC	#Participants (%)	#LLTs	Top LLTs^a^ (#Participants^b^)
**Gastrointestinal disorders**	34 (100%)	29	• Pelvic pain female (27) • Diarrhea (25) • Constipation (24) • Defecation urge painful (24) • Dyschezia (24)
**Musculoskeletal and connective tissue disorders**	34 (100%)	28	• Low back pain (28) • Defecation urge painful (24) • Scapula pain (15)
**Reproductive system and breast disorders**	34 (100%)	18	• Endometriosis-related pain (27) • Pelvic pain female (27) • Breast tenderness (22) • Painful periods (22)
**General disorders and administration site conditions**	34 (100%)	14	• Fatigue (27) • Increased appetite (21) • Decreased appetite (19)
**Nervous system disorders**	33 (97%)	26	• Headache (18) • Brain fog (15) • Dizziness (15)
**Renal and urinary disorders**	29 (85%)	8	• Pelvic pain female (27) • Incomplete urination (13) • Urination frequency of (11)
**Vascular disorders**	28 (82%)	9	• Dizziness (15) • Migraine (11) • Hot flushes (9)
**Metabolism and nutrition disorders**	26 (26%)	4	• Increased appetite (21) • Decreased appetite (19) • Food craving (15)
**Psychiatric disorders**	22 (65%)	10	• Anger (15) • Brain fog (15) • Anxiety (8) • Depression (8)
**Respiratory, thoracic and mediastinal disorders**	20 (59%)	12	• Smell alteration (7) • Chest pain (6) • Rib pain (4) • Shortness of breath (4)
**Cardiac disorders**	18 (53%)	6	• Dizziness (15) • Chest pain (6) • Shortness of breath (4)
**Skin and subcutaneous tissue disorders**	15 (44%)	11	• acne (11) • Touch sensitivity increased (4) • Hair Loss (2) • Numbness in hands, forearms, elbows (2) • Vaginal itching (2)
**Immune system disorders**	9 (26%)	6	• Multiple allergies (5) • Nasal allergy (3) • Asthma (2)
**Infections and infestations**	8 (24%)	5	• Sinusitis (3) • Eye infection (2) • Gum infection (2)
**Eye disorders**	6 (18%)	7	• Blurred vision (2) • Eye infection (2) • Eye allergy (1) • Eye pain (1) • Sensation of pressure in eye (1) • Sjogren’s (1) • Visual disturbances (1)
**Ear and labyrinth disorders**	4 (12%)	2	• Sound sensitivity increased (3) • Ear pain (2)
**Endocrine disorders**	1 (3%)	1	• Small fiber neuropathy (1)

^a^In cases where multiple LLTs share the same frequency as the third most common term, all tied LLTs are included;

^b^Number of participants who tracked each LLT.

### Consistency of symptom value distributions across cycles and patients

**Fig. 2:**
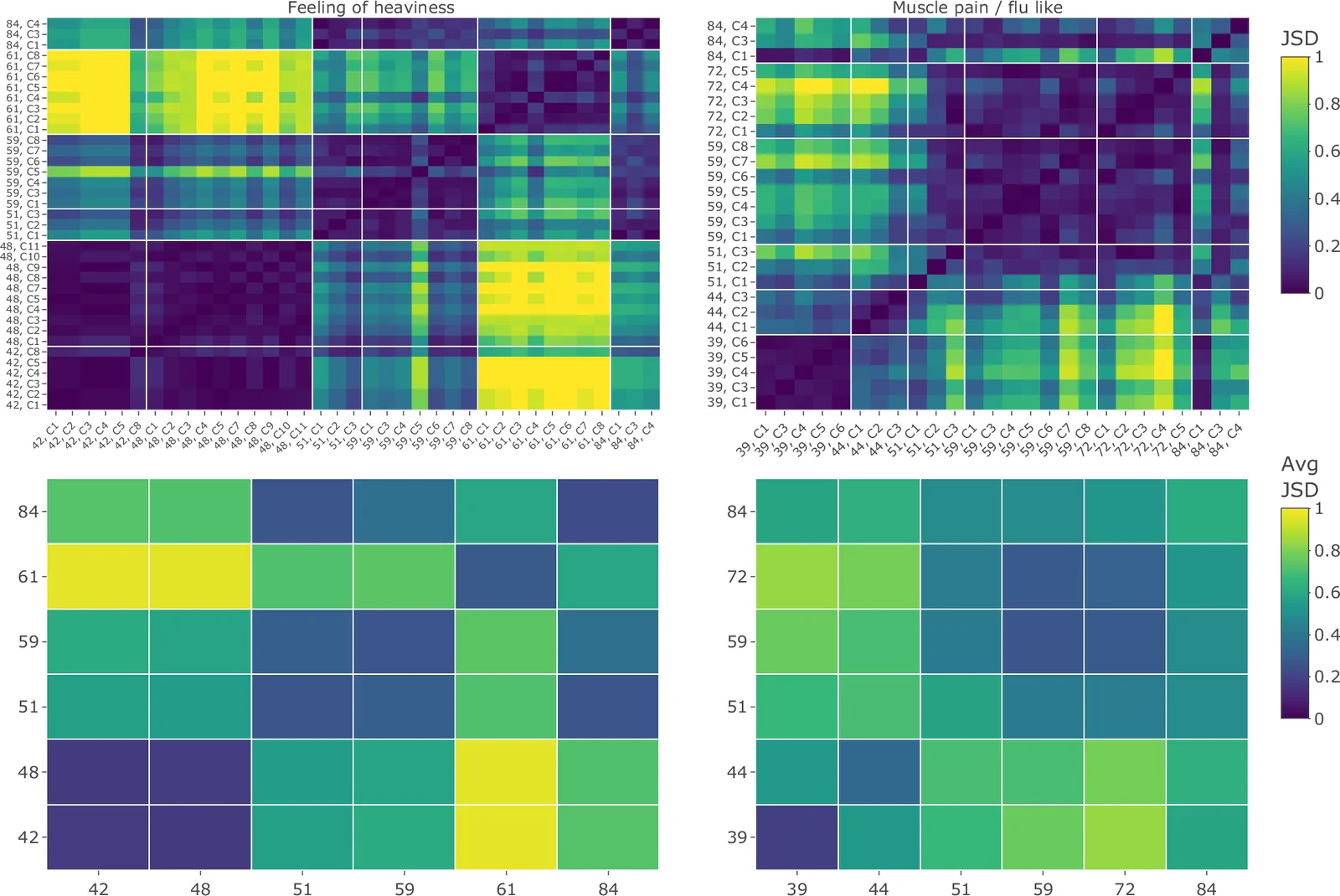
Within- and between-patient divergence of symptom-intensity distributions across menstrual cycles. Pairwise Jensen–Shannon divergence (JSD) computed between distributions of daily-reported intensity scores per cycle, for two representative symptoms: feeling of heaviness and muscle pain/flu-like symptoms. *Top row:* Cycle-level JSD matrices: each cell represents the JSD between intensity distributions in a pair of menstrual cycles, with cycles grouped by patient and ordered chronologically within patient; block-diagonal regions therefore correspond to within-patient cycle pairs and off-diagonal regions to between-patient cycle pairs. *Bottom row:* Patient-level JSD matrices: each off-diagonal cell is the mean of the square root of cycle-pair JSDs between the cycles of two patients, and each diagonal cell is the mean of square root of within-patient cycle-pair JSD. Low JSD indicates similar intensity distributions; high JSD indicates divergent distributions.

We computed pairwise Jensen–Shannon divergences (JSD)^56^ between per-cycle symptom-intensity distributions (Methods). Figure 2 shows the resulting cycle-level matrix (cycles grouped by patient, ordered chronologically within patient) and the patient-level matrix obtained by averaging cycle-pair JSDs across all cycles of each patient dyad, for two representing symptoms. For the symptom feeling of heaviness, two clusters of patients show both low within-patient divergence (consistent reporting across cycles) and low between-patient divergence (similar distributional profiles): patients 42 and 48, and patients 51, 59, and 84. Patient 61, by contrast, shows low within-patient divergence but high divergence from all other patients, indicating consistent self-reporting against an idiosyncratic personal baseline. For muscle pain/flu-like symptoms, patients 59 and 72 form a comparable within- and between-patient consistency pair. Patients 39 and 44 show consistent within-patient reporting on profiles distinct from the rest of the cohort.

Overall, within-participant JSD values were significantly lower than between-participant values in 30 of 33 analyzed symptoms (91%) (Supplementary Data 3). Symptom distributions are thus more consistent within individuals than across them, motivating a participant-level approach to symptom-trajectory analysis.

### Patient-level analysis—representative example (Patient ID 59)

**Fig. 3:**
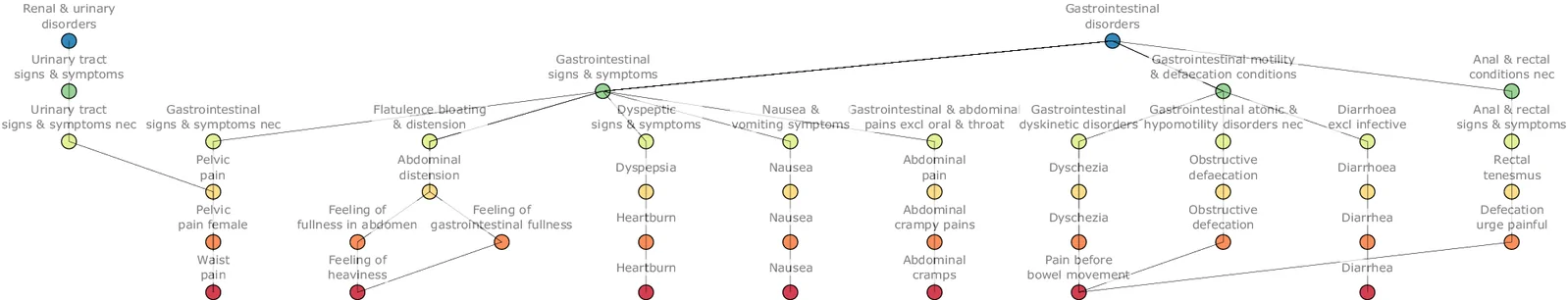
Illustration of 7 out of the 26 symptoms tracked by Patient 59, categorized according to the MedDRA hierarchy. This visualization provides an ontological representation of the patient’s symptom profile, structured by meaning and clinical domain rather than time. Dots are color-coded to represent different MedDRA hierarchy levels as follows: participant-defined symptom name, as entered and displayed in the app – red, LLT – orange, PT – yellow, HLT – light green, HLGT – green, and SOC – blue.

To further examine symptom presentation at the individual patient level, we present a representative participant from the study cohort. This example illustrates the range of symptom types monitored by the participant and shows how longitudinal tracking enables detailed characterization of symptom dynamics and overall disease burden. Patient 59 tracked 26 symptoms for 256 days with a 96.9% engagement rate, covering 8 menstrual cycles. The average menstrual cycle length was 31.8 days. Figure 3 presents 7 of her app-tracked symptoms (shown at the bottom) along with their five ancestral MedDRA levels. It demonstrates how the diverse range of symptoms can be classified into body systems, offering insight into the multisystemic nature of endometriosis. For instance, “waist pain” is categorized under both ‘Gastrointestinal Signs and Symptoms’ and ‘Renal and Urinary Disorders’ at the SOC level (see Supplementary Data 2 for the full set of symptoms for all participants, classified according to the MedDRA hierarchy).

**Fig. 4:**
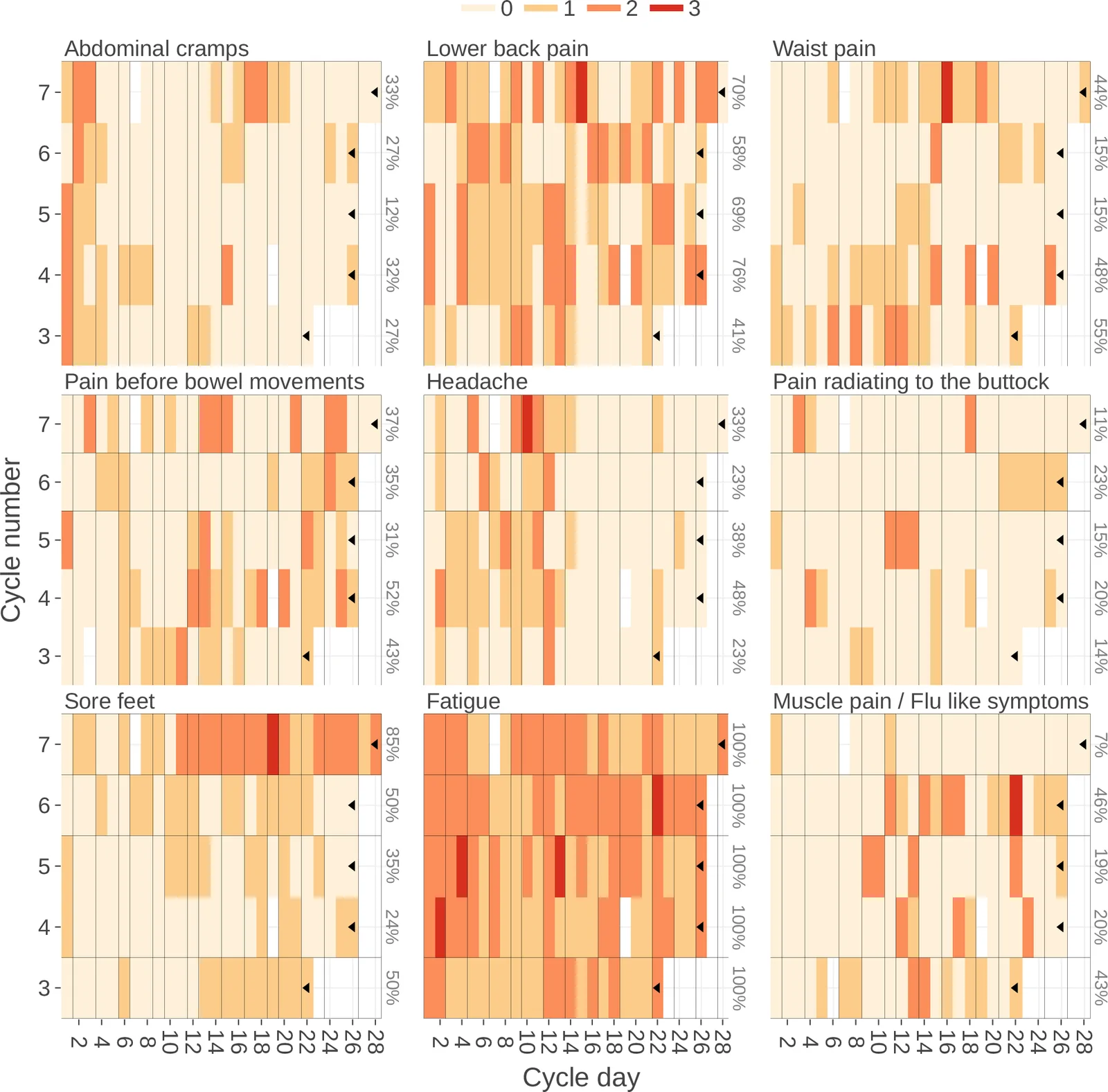
Plots showing 9 of the 26 symptoms monitored by Patient 59 (noted above each heatmap), focusing on 5 consecutive cycles out of 8 (cycles 3–7) to highlight insights from long-term tracking. All symptoms are measured on a 0–4 scale; however, for these symptoms, this patient only reported intensities ranging from 0 to 3. Black triangles mark the last day of the cycle. White days indicate a missing report, while white days following the black triangles represent days that fall outside the actual length of that cycle. The numbers to the right of each cycle represent the symptom burden, defined as the percentage of symptomatic days out of reported (non-missing) days for each symptom within that cycle.

Figure 4 presents nine representative symptoms of Patient 59 over five consecutive cycles (cycles 3–7), measured on a 0–4 scale. Although this patient tracked symptoms across eight full cycles, we chose to present cycles 3 through 7 to avoid overwhelming the visualization and because cycle 1 was unusually long, which would have affected the consistency and clarity of the overall presentation. Monitoring symptoms throughout the entire hormonal cycle across multiple cycles, provides valuable insights into how symptoms evolve over an extended period of time. For example, abdominal cramping was most prominent during menstruation, ovulation, and premenstrual syndrome (PMS), whereas lower back pain appeared more persistent throughout the cycle. Sore feet worsened in the last cycle, and fatigue is identified as the most impactful symptom, as it persists throughout the entire cycle, varying in intensity.

**Fig. 5:**
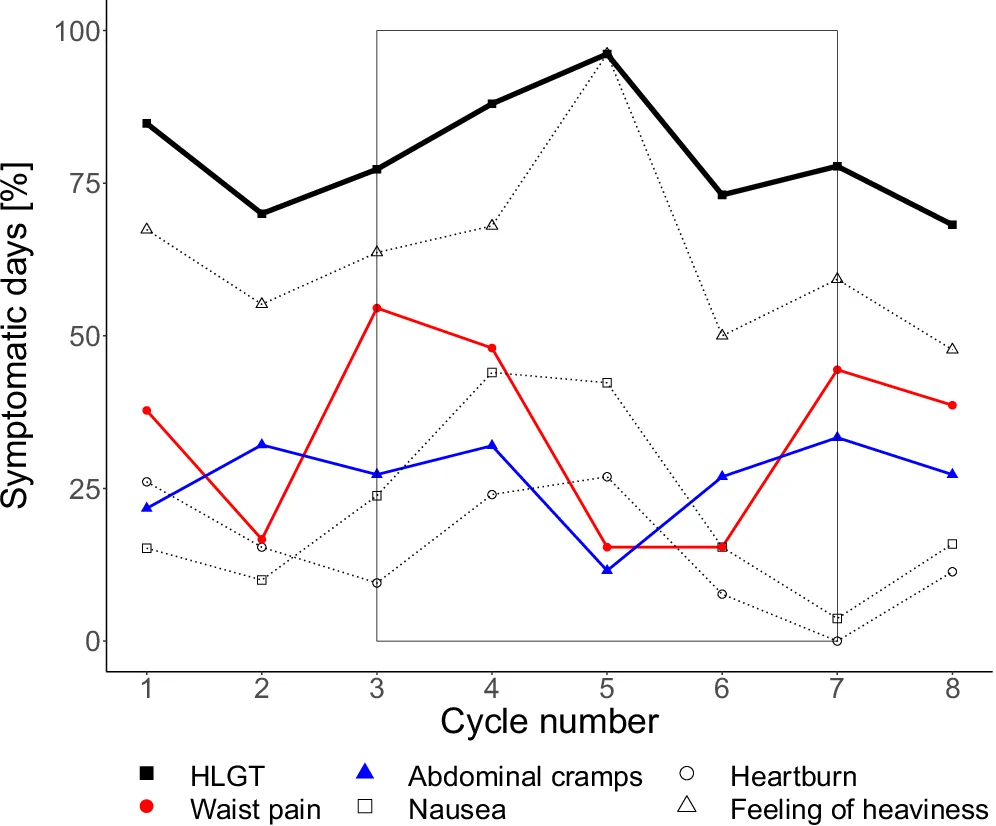
Percentage of symptomatic days for Gastrointestinal signs and symptoms at the HLGT level (i.e., symptom group burden) along with their contributing symptoms at the app level of Patient 59 (i.e., symptom burden). The black solid line represents the HLGT symptom group burden, reflecting the combined contribution of all five descendant symptoms. Solid, colored lines denote symptoms included in Fig. 4, while dotted gray lines indicate symptoms not shown in Fig. 4. Percentages are calculated using reported (non-missing) days only. The gray rectangle highlights cycles 3–7, which are presented in Fig. 4 (the MedDRA hierarchy for Gastrointestinal signs at the HLGT level is fully presented in Fig. 2).

In Fig. 4, the numbers to the right of each plot indicate the percentage of symptomatic days for each symptom within a given cycle, defined as the proportion of reported days with severity above 0, providing a quantitative measure of *symptom burden* over time. Figure 5 extends this analysis by grouping symptoms according to MedDRA’s HLGT category to evaluate *symptom group burden*; for instance, within the HLGT category of Gastrointestinal signs and symptoms (as detailed in the MedDRA hierarchy presented in Fig. 2). Patient 59 experienced 12%-33% of symptomatic days per cycle due to “abdominal cramps” and 15%-55% due to “waist pain”. However, when considering all her Gastrointestinal signs and symptoms at the HLGT level collectively (i.e., abdominal cramps, waist pain, heartburn, nausea, and feeling of heaviness), the percentage of symptomatic days rises dramatically to 75%–100%. This higher percentage represents the *symptom group burden* for the HLGT category of Gastrointestinal signs and symptoms, reflecting the persistent and widespread nature of symptoms throughout the cycle. While a single symptom may appear sporadically, the combined presence of multiple symptoms suggests that Patient 59 may suffer from continuous gastrointestinal discomfort nearly every day of the cycle. This approach highlights the importance of a comprehensive clinical perspective: evaluating symptoms in isolation underestimates the true impact of the disease, whereas category-level analysis reveals the full extent of symptom and disease burden on participant’s quality of life.

## Discussion

This study establishes the feasibility of a patient-tailored, mobile-based approach to long-term daily symptom tracking in endometriosis. Using data collected from 34 individuals over periods of up to 12 months, we demonstrate how individualized symptom reporting can be structured and scaled to capture the complexity and multisystemic nature of the disease. The high median number of symptoms per patient (24.5 [19.25, 36.5]) and the extensive number of affected SOCs (10 [9,12]), many extending far beyond the pelvic region, further underscore the systemic nature of endometriosis^3,28,46,57^.

To guide the quantification and interpretation of individualized symptom dynamics in endometriosis, we propose a conceptual framework that introduces key terms: symptom trajectory, symptom burden, symptom group burden, and disease burden. Using a patient-tailored symptom tracking approach, participants frequently reported both well-documented and under-recognized symptoms, providing insights into the heterogeneous and multisystemic nature of endometriosis. Consistent with existing literature, commonly reported symptoms included lower back pain, fatigue, and dysmenorrhea^58–62^. In addition, participants tracked a broader range of symptoms and comorbidities than standardized instruments typically capture, including eye allergies, cold sensitivity, Sjogren’s symptoms (neck/throat/eyes), genital herpes, ear pain, vocal cord problems, mouth ulcers, and more. The individualized structure of the tracking approach also allowed participants to define symptoms using their own terminology and distinctions. Participants emphasized that this fostered a sense of ownership and relevance, which appeared, based on informal feedback, to enhance engagement. For example, they often distinguished between specific pain types (e.g., left vs. right pelvic pain, “stabbing” vs. “dull”)^63^ and separated similar experiences, such as pain at different stages of intercourse, even when these mapped to the same MedDRA LLT. This underscores the uniqueness of each patient’s disease profile. Importantly, the inclusion of these symptoms does not establish them as endometriosis-attributable manifestations. Given the clinical heterogeneity of endometriosis, we deliberately did not restrict symptom capture a priori. The breadth of symptoms documented here is consistent with prior reports of extensive comorbidity profiles in endometriosis^11^ and highlights candidate symptoms for targeted follow-up. These findings warrant further investigation in larger and more representative populations. More broadly, they highlight the importance of systematically exploring the full spectrum of symptoms and the underlying pathophysiological mechanisms of endometriosis. A deeper understanding of these mechanisms and their multisystemic manifestations could support the development of more targeted treatments and, ultimately, improve patients’ quality of life.

To enable structured analysis of these highly individualized symptom reports, we integrated digital symptom tracking with the MedDRA hierarchy, mapping participant-reported symptoms to standardized medical terms. Its hierarchical structure enables clinical synthesis by organizing symptoms into broader categories, allowing for structured analysis of systemic involvement. While symptoms were standardized for aggregate analysis, the original inputs remained deeply personal and participant-driven. We view MedDRA standardization not as limiting personalization, but as a bridge between patient-reported data and clinical frameworks, supporting both individualized care and population-level insight into the multisystemic nature of endometriosis.

Endometriosis patients face a significant challenge in effectively communicating the fluctuations and severity of their symptoms throughout the hormonal cycle. To address this, we introduce two analytical concepts, symptom burden and symptom trajectory, which together provide a comprehensive overview of symptom dynamics, severity, and persistence over time. Consistent with the distribution-level JSD analysis, demonstrating greater within-patient than between-patient similarity, the use of personalized symptom scales preserves sensitivity to individual symptom dynamics and supports within-participant longitudinal analysis. It does, however, limit direct comparison of absolute severity across individuals. While this study did not assess clinical outcomes directly, these concepts lay a foundation for future tools aimed at improving disease monitoring and individualized care.

By defining symptom burden in terms of symptomatic days (i.e., days with symptom scores above zero), we capture the full spectrum of pain and discomfort across the cycle. Notably, what qualifies as a symptomatic day is determined based on each participant’s personalized pain scale, ensuring that the measure reflects individual variations in pain sensitivity and perception. To assess disease burden comprehensively, we analyze both individual symptoms and aggregated symptom groups as defined by the MedDRA hierarchy. This dual approach captures the broader multisystemic impact of endometriosis, recognizing that improvement in a single symptom may not equate to meaningful relief if others persist or worsen. Ultimately, the goal is to reduce disease burden by increasing the proportion of asymptomatic days. Therefore, it is sometimes necessary to examine symptom groups as categorized by MedDRA, rather than analyzing only individual symptoms, in order to estimate the cumulative impact of the disease on overall quality of life.

Analyzing symptom trajectories over extended periods supports investigation of symptom dynamics and variability at the individual level. In addition, the distribution-level JSD analysis demonstrated that distributional consistency is stronger within than between individuals, a pattern that motivates patient-level analysis of symptom trajectories. Notably, inter-patient similarity was symptom-dependent rather than globally preserved across all symptoms. This analysis does not incorporate temporal information within the menstrual cycle and therefore does not directly assess phase-specific dynamics.

We conducted an in-depth single-case analysis of a representative participant, supplemented by a cross-participant comparison to identify shared structural characteristics. Future research may involve longitudinal analysis of individual cases to identify cyclical patterns, followed by comparisons across participants to detect shared or divergent trajectories. This bottom-up approach, from individual patterns to population-level insights, may uncover hidden symptom dynamics and support more effective, personalized disease management.

This study has a few limitations. First, throughout the study, our research team maintained close communication with participants, offering guidance on symptom and scale definitions and conducting monthly check-ins. While these interactions may be perceived as interventions, potentially influencing symptom reporting, they were essential for sustaining engagement and ensuring the accuracy of long-term daily data collection.

Second, daily symptom tracking itself poses challenges. Participants sometimes skipped entries on asymptomatic days to avoid thinking about pain or simply due to forgetfulness, and on symptomatic days because the symptoms were too overwhelming. To minimize dropouts and ensure data completeness, we sent reminders when participants had not logged symptoms for more than three consecutive days and allowed retrospective data entry for up to seven days. These strategies, along with regular check-ins, likely contributed to the high engagement rate observed in the study. Accordingly, the observed engagement reflects a supported research setting designed to enable complete longitudinal data collection and may not directly generalize to real-world use without similar support structures. In addition, the study was not designed to determine the minimum duration of tracking required to capture meaningful symptom dynamics. Multiple consecutive cycles are likely necessary; however, defining the optimal tracking duration remains an important direction for future research.

Third, we did not recruit a control group to track monthly, potentially hormone-related symptoms and, as a result, cannot make relative claims about the symptom burden of endometriosis compared to healthy individuals or those with other chronic conditions. Beyond the technical challenges such control groups present—e.g., healthy individuals are less likely to consistently monitor symptoms, especially mild or non-disruptive—this study was primarily designed to demonstrate the value of personalized symptom tracking, rather than to compare endometriosis to other conditions. Moreover, this cohort is small and not intended to be representative of the broader endometriosis population. Accordingly, our observations should not be interpreted as estimates of symptom prevalence. Future research may incorporate matched control groups to better identify which symptom patterns are unique to endometriosis.

Finally, life circumstances occasionally disrupted symptom tracking. Common disruptions included vacations, pregnancy, endometriosis surgery, and major life events such as job loss, divorce, or extended travel. Moreover, the study took place in Israel between 2023 and 2025, a period marked by war and national stress, which further contributed to interruptions in tracking. Notably, no participants left the study due to initiation of hormonal treatment.

In conclusion, this study underscores the multifaceted nature of endometriosis and emphasizes the need for individualized symptom monitoring to better understand the disease profile, progression and burden. Across our dataset, we observed substantial variability in symptom profiles, trajectories, and overall disease burden. A detailed case study illustrates how symptom dynamics can differ across cycles, revealing both persistent and episodic symptoms. Our personalized symptom tracking approach, structured using the MedDRA hierarchy, provides a framework for capturing the complexity of longitudinal symptom dynamics. By introducing new terminology—namely, symptom trajectory, symptom burden, and disease burden—we propose a conceptual framework for understanding the broader impact of endometriosis at both the symptom and systemic levels. We believe this framework can be extended beyond endometriosis to other complex, multisystemic or hormone-related conditions, where symptom heterogeneity and fluctuating trajectories challenge diagnosis and treatment. Personalized, structured symptom tracking has the potential to uncover hidden dynamics, guide targeted interventions, and ultimately support more informed clinical decision-making.

## Methods

This prospective study involved endometriosis patients monitoring a self-defined set of symptoms and scales daily over a period of up to 12 months using a custom-developed mobile app. Home ovulation test kits were used to determine hormonal stages for each patient, and at the end of each hormonal cycle, cumulative symptom reports were generated and shared with participants to encourage continued engagement and provide patients with the option to share their data with their care team.

### Ethical approval

This study was approved by the Haifa University ethical committee (Reference number 367/23). All participants provided written informed consent prior to participation in the study.

### Recruitment of participants

Participants were recruited through multiple channels, including Facebook groups of private health professionals (e.g., nutritionists, physiotherapists), Instagram accounts of influencers whose primary audience consists of women of reproductive age (in fields such as fashion, food, wellness, and fitness), and the clinics of physicians specializing in endometriosis. Participants were not compensated for their participation in the study.

### Study population

Participants were eligible if they had a formal diagnosis of endometriosis made by a recognized endometriosis specialist, in accordance with Israeli clinical guidelines, which do not require surgical confirmation. Specialists were identified based on the list published by the Endometriosis Foundation of Israel (https://endoisrael.org/clinics/). Additionally, participants were eligible if they were not using any form of hormonal treatment during the study period; had spontaneous (non-hormonally regulated) menstrual cycles with typical cycle lengths between 20–35 days prior to enrollment; had not entered menopause; and, if they had undergone endometriosis surgery, at least three months had passed since the procedure.

### Study design

Prior to the start of the study, interested individuals participated in an initial screening call with the research team. During this call, the study objectives were explained, consent and legal documentation were reviewed, and data privacy and confidentiality measures were discussed. Each participant’s symptoms and overall condition were assessed to determine eligibility based on the study’s inclusion criteria.

If eligible and willing to participate, individuals first completed a medical questionnaire, followed by a second interview to define their personalized symptom profile and select appropriate intensity scales. With our guidance, participants identified the symptoms most relevant to their experience and chose how to rate them, typically using 0–10 or 0–4 numerical scales, representing a range from ‘symptom-free’ to ‘symptom at its highest intensity’ ^46^.

Following the initial setup, participants used the app to track their symptoms daily over a two-week familiarization period. This allowed them to get comfortable with the app’s structure and features. At the end of this period, participants were given the opportunity to revise their symptom list or adjust the rating scales associated with each symptom. Next, they defined verbal anchors to describe what each number on the scale represented in terms of intensity or impact. This process allowed for a more objective and consistent interpretation of symptom ratings while preserving individual nuance and relevance in daily tracking. Additionally, all participants tracked their general physical and emotional condition daily using a 1–10 or 1–4 scale, with personalized verbal anchors defined in a manner similar to the pain scales, where 1 represented the worst day and the highest value represented the best day. This personalized scaling approach differs from validated instruments such as the EHP-30^64,65^, which are administered at discrete intervals (e.g., every few months) to assess quality-of-life impact, or experience sampling method (ESM)^50^ approaches that capture high-frequency data over short study windows (e.g., multiple reports per day over several days) using predefined symptom lists. In contrast, our framework is designed for sustained, longitudinal daily tracking across extended periods. Participants also tracked any medications or treatments used to manage pain. A free-text option allowed them to note anything additional that occurred on a given day (further details on daily data monitoring and personalization can be found in the Supplementary Note 2). Once each participant finalized their symptom list and personalized scales, these settings were locked for the duration of the study to ensure consistency in data collection; however, participants could add new symptoms if they emerged during the study period or remove symptoms that were no longer relevant. This time point was considered the official start date of the study period for that participant. From that point on, participants tracked their symptoms daily, reporting a numerical value for each symptom in their personalized list.

To monitor hormonal stages, participants were asked to log whether they were menstruating (yes/no), and to rate menstrual bleeding volume using a personalized 0–4 scale^63^. Each participant defined the levels of bleeding based on their own experience and product usage (e.g., tampons, pads, panty liners). To track ovulation, they used a commercial ovulation kit at home, starting on day 9 and continuing through day 20. If no rapid increase in luteinizing hormone (LH) levels (i.e., peak) was detected, they were instructed to test until one appeared, then monitor for a drop in LH levels over the following 2–3 days. This approach aimed to enhance the accuracy of hormonal stage identification. This information was collected to provide temporal reference points for each participant’s data but was not incorporated into the analyses presented in the current study.

To support sustained data collection, the research team contacted participants who failed to submit data for more than three consecutive days to check in, ensure their well-being, and provide a reminder. The app allowed participants to backfill symptom data for up to seven days to accommodate occasional lapses. Some participants took planned breaks (e.g., due to vacations or personal reasons), which they communicated in advance. For analysis, we concatenated each participant’s active reporting periods and excluded these gaps to maintain continuity in the data. To quantify engagement, we classified a participant-day as “engaged” if at least 50% of tracked symptoms were logged, excluding non-symptom entries (e.g., medications, alternative-medicine treatments, and sick-day annotations). Participant-level engagement was defined as the proportion of engaged days across the participant’s total study duration.

At the end of each hormonal cycle, we generated an updated cumulative symptom report for each participant, summarizing all data collected from the beginning of the study up to that point. After each report was sent, we conducted a remote video interview with the participant to review the findings together, ensure the report accurately reflected their experience, and encourage continued participation.

### Data collection through a digital app

To simplify the documentation process and enhance engagement and adherence, we developed a custom tracking app to collect patient-reported data. The app included a calendar view designed to support user engagement and provide visual feedback. This calendar used distinct visual markers to indicate various event types and levels of data completeness, such as days with reported menstrual bleeding; days designated for ovulation testing; days with complete data entries; days with partial data entries; and days with no submitted data (further details on the digital app can be found in the Supplementary Note 2).

### Database and privacy

All participants’ data were stored in a secure, cloud-based PostgreSQL database using a dual-schema structure to ensure privacy. Personally identifiable information was stored separately from symptom data and was accessible only to authorized researchers. Each participant was assigned a unique identifier, which linked their anonymized symptom records for analysis. Further details on data privacy and management are provided in the Supplementary Note 1.

### Symptom classification

To enable standardized analysis, we mapped the individualized 228 symptoms and conditions to the Medical Dictionary for Regulatory Activities (MedDRA^®^)^54,55^, an internationally recognized hierarchical medical terminology developed under the auspices of the International Council for Harmonisation of Technical Requirements for Pharmaceuticals for Human Use (ICH; MedDRA^®^ trademark is registered by ICH). We selected MedDRA due to its detailed structure, broad clinical scope, and compatibility with symptom classification across multiple organ systems. Its hierarchical framework, from Lowest Level Terms (LLTs) to System Organ Classes (SOCs), allowed us to analyze symptoms across different levels of granularity and to compare our findings with other clinical and regulatory datasets. Mapping was manually performed by the research team, selecting the LLT closest to the participant-defined description. When mapping was not straightforward, decisions were discussed with the clinical advisor and resolved based on the clinical context provided during onboarding. In cases where multiple LLTs were considered clinically appropriate, one-to-many mappings were retained. Each symptom was then positioned within the full MedDRA hierarchy, progressing from LLT to Preferred Term (PT), High-Level Term (HLT), High-Level Group Term (HLGT), and ultimately to the SOCs. For example, as shown in Fig. 3, the participant chose to monitor a symptom described as “feeling of heaviness” (i.e., abdominal heaviness). This term was linked to two LLTs: “feeling of fullness in abdomen” and “feeling of gastrointestinal fullness”. Both map to the same PT: “abdominal distension”, under the HLT: “flatulence, bloating and distension”, HLGT: “gastrointestinal signs and symptoms”, and ultimately the SOC: “gastrointestinal disorders”. The complete symptom-to-MedDRA mapping table is provided as a Supplementary Data 2. A detailed description of the MedDRA mapping procedure is provided in the Supplementary Note 3.

### Data analysis

Data analysis was conducted in two stages: (i) a distribution-level similarity analysis to assess consistency of symptom reporting across cycles and participants, and (ii) case study of longitudinal analyses to characterize symptom dynamics over time.

For the first stage, we assessed the consistency of symptom reporting across menstrual cycles by analyzing the distribution of symptom severity values at the cycle level. For each symptom, we computed, for each patient and each cycle, the empirical distribution of reported severity values (0–4). Cycles with more than 10% missing daily reports were excluded, and patients with fewer than three cycles were removed from the analysis. Distributional differences were quantified using the Jensen–Shannon divergence (JSD), a symmetric, bounded dissimilarity measure derived from the Kullback–Leibler divergence, taking values in [0, 1] under log base 2, with 0 indicating identical distributions and 1 indicating maximal divergence^56^. Pairwise JSD values were computed between all cycles across all patients, including within- and between-patient comparisons. To derive patient-level divergence, square root JSD values were averaged across all pairwise cycle comparisons between two patients^66^. For each symptom, we compared within- and between-participant JSD distributions using a two-sided Mann-Whitney U test. The resulting p-values were corrected for multiple comparisons using the Benjamini–Hochberg false discovery rate (FDR) procedure^67^ yielding q-values; symptoms with *q* < 0.05 were considered statistically significant.

For the second stage, we performed within-participant longitudinal analyses using three complementary approaches. First, we presented *symptom trajectories*, displaying the daily participant-reported appearance and severity of individual symptoms across several consecutive cycles. This approach highlights fluctuations in symptom intensity over time and enables comparisons across cycles. It helps identify specific periods that may require targeted interventions and assess changes following interventions. Next, we introduced the concept of *symptom burden*, which quantifies the percentage of symptomatic days out of reported days within each cycle, defined here, as proof of concept, as any day on which a symptom was reported with a value above 0. To reduce sensitivity to participant-specific scale definitions, burden was operationalized using a binary threshold (>0), making it invariant to differences in scale range (e.g., 0–4 vs 0–10). All metrics were computed within participants and across observed days only, thereby limiting the impact of intermittent missing data and avoiding assumptions of cross-participant comparability. Accordingly, these measures are designed to capture within-subject temporal dynamics rather than absolute severity differences between individuals. While a uniform threshold was used here, alternative thresholds may be explored in future work to better reflect individual symptom significance. As we aim to quantify the overall impact of the disease, it is sometimes necessary to assess the cumulative burden imposed by a cluster of related symptoms. We therefore defined *symptom group burden*, for a given MedDRA level, where a day is considered ‘symptomatic’ if at least one symptom within that group is symptomatic (here, had a value above 0). As we move higher up the MedDRA hierarchy, we gain a broader perspective on the distribution of symptomatic versus asymptomatic days across grouped symptoms. When aggregating all groups and symptoms together, we refer to the overall *disease burden*. These metrics are intended for within-participant longitudinal analysis and are not directly comparable across individuals due to the use of personalized symptom scales and definitions.

## Supplementary information

Supplementary Information

Supplementary Data 1

Supplementary Data 2

Supplementary Data 3

## Data Availability

Data collected and generated during this study are available via the PhysioNet^68^ platform (https://physionet.org/content/ki-endolist/1.0.0/), including participant-reported symptoms, a comprehensive symptom-to-MedDRA mapping table, and participant-level mappings^69^.
