# Supplementary material for: Patient-tailored symptom tracking in endometriosis: a framework to explore disease variability and burden
Source: NPJ Digit Med. 2026 Jul 17;9:888. doi: 10.1038/s41746-026-02965-z (PMC13759499; doi:10.1038/s41746-026-02965-z)
Supplement: Supplementary file 1 [file 41746_2026_2965_MOESM1_ESM.docx]

**Patient-tailored symptom tracking in endometriosis: a framework to explore disease variability and burden**

**Supporting information**

Tamar Zelovich^1^, Alon Zelovich^1^, Shaked Feiglin^1^, Vered Klaitman-Mayer^2,3^, Noémie Elhadad^4^, Ronya Rubinstein^1^, Ronit Endevelt^5^, Pinchas Akiva^1^, Maytal Bivas-Benita^1^, Chen Yanover^1^

## Supplementary Tables and Figures

**Table S****1. Cohort Characteristics:** An overview of the study cohort and its characteristics

| **N** | 34 |  |  |
| --- | --- | --- | --- |
| **Marital status**^†^ |  | **Work Setting^†^** |  |
| Married | 13 (38%) | Working from office | 10 (29%) |
| In a relationship | 10 (29%) | Working from home | 5 (15%) |
| Single | 10 (29%) | Hybrid | 12 (35%) |
| Widow | 1 (3%) | Not working / Not relevant | 7 (21%) |
| Divorcee* | 1 (3%) | **Education**^†^ |  |
|  |  | High School | 6 (18%) |
| **Employment Status^**,^ ^†^** |  | Certificate Studies | 4 (12%) |
| Full time | 15 (44%) | Academic - Bachelor's degree | 18 (53%) |
| Part time | 14 (41%) | Academic - Master's degree | 6 (18%) |
| Student | 8 (24%) | **Effect on relationships**^†^ |  |
| Student job | 1 (3%) | Yes | 4 (12%) |
| Not working | 4 (12%) | No | 21 (62%) |
|  |  | Don't know | 9 (26%) |

**^†^** Number of participants (% of the study cohort), ^*^One patient is marked as both divorced and in a relationship, ^**^Two patients are identified as both students and holding full-time jobs; five patients are marked as both students and having part-time jobs; and one patient is marked as both a student and having a student job.

**Figure S1:** Age distribution among the 34 participants included in the study. Age was self-reported at enrollment. The histogram illustrates the variability in participant age within the study cohort.

**Table S****2. Study Characteristics:** A detailed per-patient breakdown of the study characteristics.

| User ID | #Symptoms | #LLT | #PT | #HLT | #HLGT | #SOC | Days in study | Engagement | #Cycles | Cycle length |
| --- | --- | --- | --- | --- | --- | --- | --- | --- | --- | --- |
| **38** | 12 | 17 | 15 | 17 | 14 | 8 | 355 | 97.2 | 12 | 28.9 |
| **39** | 19 | 19 | 19 | 19 | 16 | 9 | 240 | 95.8 | 6 | 39.2 |
| **41** | 16 | 20 | 19 | 22 | 17 | 9 | 254 | 84.6 | 9 | 27.3 |
| **42** | 28 | 29 | 25 | 28 | 19 | 9 | 220 | 88.2 | 8 | 25.1 |
| **44** | 17 | 18 | 17 | 20 | 14 | 7 | 110 | 96.4 | 3 | 31.3 |
| **47** | 43 | 41 | 37 | 38 | 25 | 13 | 49 | 73.5 | 1 | 30.0 |
| **48** | 22 | 28 | 25 | 27 | 17 | 10 | 299 | 95.7 | 11 | 27.1 |
| **49** | 21 | 27 | 25 | 29 | 18 | 10 | 53 | 98.1 | 2 | 25.0 |
| **50** | 16 | 15 | 14 | 16 | 12 | 6 | 13 | 92.3 | – | – |
| **51** | 27 | 28 | 24 | 23 | 16 | 8 | 101 | 100.0 | 3 | 28.7 |
| **52** | 22 | 24 | 24 | 26 | 17 | 10 | 215 | 94.9 | 7 | 27.4 |
| **53** | 40 | 43 | 42 | 39 | 25 | 12 | 238 | 97.5 | 7 | 33.9 |
| **59** | 26 | 31 | 29 | 30 | 22 | 13 | 256 | 96.9 | 8 | 31.8 |
| **61** | 34 | 37 | 33 | 35 | 24 | 10 | 241 | 94.2 | 9 | 26.6 |
| **62** | 41 | 41 | 38 | 41 | 28 | 12 | 193 | 96.9 | 6 | 31.5 |
| **63** | 34 | 41 | 41 | 41 | 30 | 13 | 91 | 71.4 | 3 | 28.3 |
| **64** | 43 | 43 | 42 | 40 | 25 | 10 | 229 | 68.6 | 7 | 31.6 |
| **65** | 23 | 27 | 25 | 27 | 17 | 8 | 160 | 74.4 | 6 | 26.5 |
| **66** | 35 | 40 | 36 | 40 | 27 | 12 | 186 | 74.2 | 7 | 26.4 |
| **67** | 31 | 30 | 27 | 33 | 23 | 11 | 169 | 100.0 | 4 | 40.8 |
| **69** | 41 | 45 | 42 | 48 | 34 | 13 | 62 | 100.0 | 1 | 30.0 |
| **71** | 17 | 18 | 18 | 16 | 14 | 8 | 130 | 100.0 | 5 | 25.8 |
| **72** | 34 | 37 | 35 | 36 | 26 | 14 | 138 | 100.0 | 5 | 27.4 |
| **73** | 17 | 20 | 18 | 16 | 11 | 8 | 139 | 98.6 | 5 | 27.6 |
| **74** | 60 | 68 | 66 | 67 | 44 | 16 | 65 | 56.9 | 3 | 31.0 |
| **75** | 20 | 21 | 20 | 19 | 19 | 10 | 144 | 96.5 | 5 | 26.4 |
| **77** | 37 | 38 | 35 | 39 | 27 | 13 | 88 | 84.1 | 3 | 27.3 |
| **79** | 20 | 20 | 20 | 22 | 16 | 8 | 89 | 100.0 | 2 | 44.0 |
| **80** | 38 | 42 | 40 | 35 | 23 | 11 | 101 | 68.3 | 3 | 30.7 |
| **81** | 14 | 17 | 17 | 18 | 15 | 9 | 17 | 47.1 | – | – |
| **82** | 22 | 26 | 26 | 22 | 16 | 9 | 113 | 86.7 | 4 | 26.0 |
| **83** | 23 | 26 | 26 | 24 | 21 | 11 | 111 | 36.0 | 3 | 30.0 |
| **84** | 19 | 26 | 20 | 21 | 15 | 11 | 121 | 96.7 | 4 | 27.8 |
| **87** | 56 | 58 | 56 | 45 | 30 | 14 | 122 | 87.7 | 4 | 30.0 |

**Figure S****2. Distribution of selected study-related attributes from Table 2 of the main text.** The y-axis represents the number of participants. Panels illustrate counts of distinct symptom categories represented in each participant’s tracked symptoms at the level of (A) app-defined symptoms, (B) Preferred Terms (PT), (C) High-Level Terms (HLT), and (D) High-Level Group Terms (HLGT) within the MedDRA hierarchy. Additional panels show (E) the total number of days each participant was enrolled in the study and (F) the number of complete cycles reported by each participant.

## Supplementary Notes

### Supplementary Note 1 – Data Privacy and Management

All patient data were stored in a secure, cloud-based PostgreSQL database. To ensure the privacy and security of participant information, a dual-schema structure was implemented. The first schema contained personally identifiable information (PII), including full name, email address, physical address, national ID number, and password. Each participant was assigned a unique identifier, referred to as the User_Primary_Key, which served as their internal reference throughout the entire database system. Access to this schema was strictly limited to authorized members of the research team and protected through restricted credentials. The second schema stored only de-identified symptom data and study-related entries. This schema included participants’ daily symptom and scale entries, linked solely via the User_Primary_Key, with no access to personal identifiers. This structure ensured that researchers conducting data analysis worked exclusively with anonymized data. Upon withdrawal from the study, personal data were deleted, but symptom data were retained for research purposes in accordance with Institutional Review Board (IRB) approval. If a participant chose to rejoin the study at a later date, a new User_Primary_Key would be generated and their prior data would not be linked to their new record, maintaining the separation of identity across study phases. All data handling procedures were designed to comply with applicable data protection standards and were approved by the relevant IRB.

### Supplementary Note 2 – Monitoring Daily Data and Personalization

**Personal Pain Scale:** Our research team supported the participants in developing personalized numerical scales tailored to their experience. For each symptom, participants first selected a numerical range, either 0–10 or 0–4, based on what felt most intuitive and useful to them. Next, they defined verbal anchors to describe what each number on the scale represented in terms of intensity or impact. These descriptions could be applied in one of three ways: a single set of verbal anchors used across all symptoms; separate sets of descriptions for different symptom categories, for example, fatigue-related symptoms (e.g., brain fog, flu-like symptoms, muscle weakness), pain-related symptoms (e.g., abdominal pain, bowel movement pain, lower back pain), or emotional symptoms (e.g., anger, depression); or unique descriptions for each individual symptom. This process allowed for a more objective and consistent interpretation of symptom ratings while preserving individual nuance and relevance in daily tracking.

**General Physical and Emotional Condition:** All participants were asked to track their general physical and emotional condition daily, using either a 1–10 or 1–4 scale (depending on the participant’s preference), with 1 representing the worst condition and 10 (or 4) representing feeling great. The same approach used to define personalized pain scales was applied here: the research team worked collaboratively with each participant to define the meaning of each numerical value on the scale to ensure individual relevance and improve consistency in daily reporting. Although these general condition scores are not analyzed in the current study, they were collected to support future research aimed at exploring how overall physical and emotional well-being may correlate with specific symptom patterns, for example, examining how pain versus fatigue influences general daily ratings, or whether there is a relationship between physical and emotional condition scores.

**Medications:** Each participant tracked any medications or treatments used to manage pain. This included pain medications, sleep aids, and non-pharmacological interventions such as acupuncture or physical therapy. Participants could also report sick days or other pain-related events as part of their daily monitoring.

**Data Collection Through a Digital App:** To simplify the documentation process and enhance engagement and adherence, we developed a custom tracking app to collect patient-reported data. Each day, participants were prompted to answer a core set of questions in the following order: whether it was the first day of their period (yes/no), whether they were menstruating (yes/no), the intensity of bleeding (0–4), general physical condition, and general emotional condition. This was followed by a personalized set of symptoms and medications, as well as individualized pain scales. As described in the previous section, the bleeding scale, physical and emotional condition ratings, symptom list, medications, and pain scales were all personalized for each participant.

The app also included a calendar view to support user engagement and provide visual feedback. The calendar used distinct visual markers to indicate different types of events and data completeness: a red line marked days with reported menstrual bleeding; a purple line indicated days designated for ovulation testing; a solid blue circle showed days when all required data were submitted; a light blue circle represented days with only partial data entries; and no circle indicated days with no submitted data.

Participants could enter or update data throughout the day. While the system automatically saved all entries, only the most recent value submitted for each symptom on a given day was analyzed and displayed in the monthly reports.

### Supplementary Note 3 – MedDRA Mapping Procedure

Mapping individualized symptoms to MedDRA entities followed a structured multi-step process:

1. Participant-defined symptom collection: Symptoms were first defined in the participant’s own language during structured onboarding interviews.
2. Initial LLT identification: Each participant-defined symptom was reviewed and mapped to the most appropriate MedDRA Lowest Level Term (LLT) using the MedDRA browser, as follows:
   2.1 If a participant-defined term directly matched an LLT, that LLT was selected.
   2.2 When mapping was not straightforward, the decision was discussed with a clinical advisor (OB-GYN specialist on the research team) and resolved based on the clinical context provided during onboarding.
   2.3 If more than one LLT was considered clinically appropriate, one-to-many mappings were retained.
3. Hierarchy propagation: Once an LLT was selected, its corresponding Preferred Term (PT), High-Level Term (HLT), High-Level Group Term (HLGT), and System Organ Class (SOC) were determined according to the MedDRA hierarchy, unless it was considered clinically irrelevant in the context of endometriosis. For example, pain during intercourse was not classified under the Psychiatric Disorders SOC, despite its placement in the standard hierarchy.

## Supplementary Data Files

**Supplementary Data 1 – Symptom’s Frequencies:** presents the frequency of tracked symptoms across all system levels in the MedDRA hierarchy (i.e., App Name, LLT, PT, HLT, HLGT, and SOC).

**Supplementary Data 2 – Symptom Classification per Patient (MedDRA Hierarchy):** contains a comprehensive list of all symptoms tracked by each patient, along with their corresponding hierarchical classification based on the MedDRA system. Each row represents a single symptom reported by a specific patient. It is important to note that, in addition to their personal symptoms, each patient also monitors general physical state, general emotional state, ovulation values, and menstrual status (yes/no). **Supplementary Data 2** includes only the patient’s personal list of symptoms that have a corresponding MedDRA classification.

**Supplementary Data 3 – Symptom-Level JSD Analysis:** provides median and IQR of within- and between-participant Jensen–Shannon divergence (JSD) values, calculated from cycle-level symptom severity distributions, together with the corresponding statistical comparisons and FDR-adjusted q-values.
